# A game-theoretic model of lymphatic filariasis prevention

**DOI:** 10.1371/journal.pntd.0010765

**Published:** 2022-09-22

**Authors:** Jan Rychtář, Dewey Taylor

**Affiliations:** Department of Mathematics and Applied Mathematics, Virginia Commonwealth University, Richmond, Virginia, United States of America; University of Utah, UNITED STATES

## Abstract

Lymphatic filariasis (LF) is a mosquito-borne parasitic neglected tropical disease. In 2000, WHO launched the Global Programme to Eliminate Lymphatic Filariasis (GPELF) as a public health problem. In 2020, new goals for 2030 were set which includes a reduction to 0 of the total population requiring Mass Drug Administrations (MDA), a primary tool of GPELF. We develop a mathematical model to study what can happen at the end of MDA. We use a game-theoretic approach to assess the voluntary use of insect repellents in the prevention of the spread of LF through vector bites. Our results show that when individuals use what they perceive as optimal levels of protection, the LF incidence rates will become high. This is in striking difference to other vector-borne NTDs such as Chagas or zika. We conclude that the voluntary use of the protection alone will not be enough to keep LF eliminated as a public health problem and a more coordinated effort will be needed at the end of MDA.

## Author summary

We adapt a compartmental ODE model of lymphatic filariasis (LF) transmission and focus our attention on what happens after Mass Drug Administrations (MDA) is terminated. We add a game-theoretic component to the model and study whether LF transmission can be substantially interrupted by voluntary use of personal protection strategies such as using insect repellents. We identify optimal voluntary protection levels and demonstrate that LF incidence rates will become too high.

## 1 Introduction

Lymphatic filariasis (LF), also known as elephantiasis, is a mosquito-borne parasitic disease caused by microscopic filarial roundworms *Wuchereria bancrofti*, *Brugia malayi* and *Brugia timori* [1]. The roundworms are transmitted to humans by mosquitoes of the genera *Aedes, Anopheles, Culex* and *Mansonia* [1]. LF is one of the leading causes of chronic disability worldwide [2].

**Fig 1:**
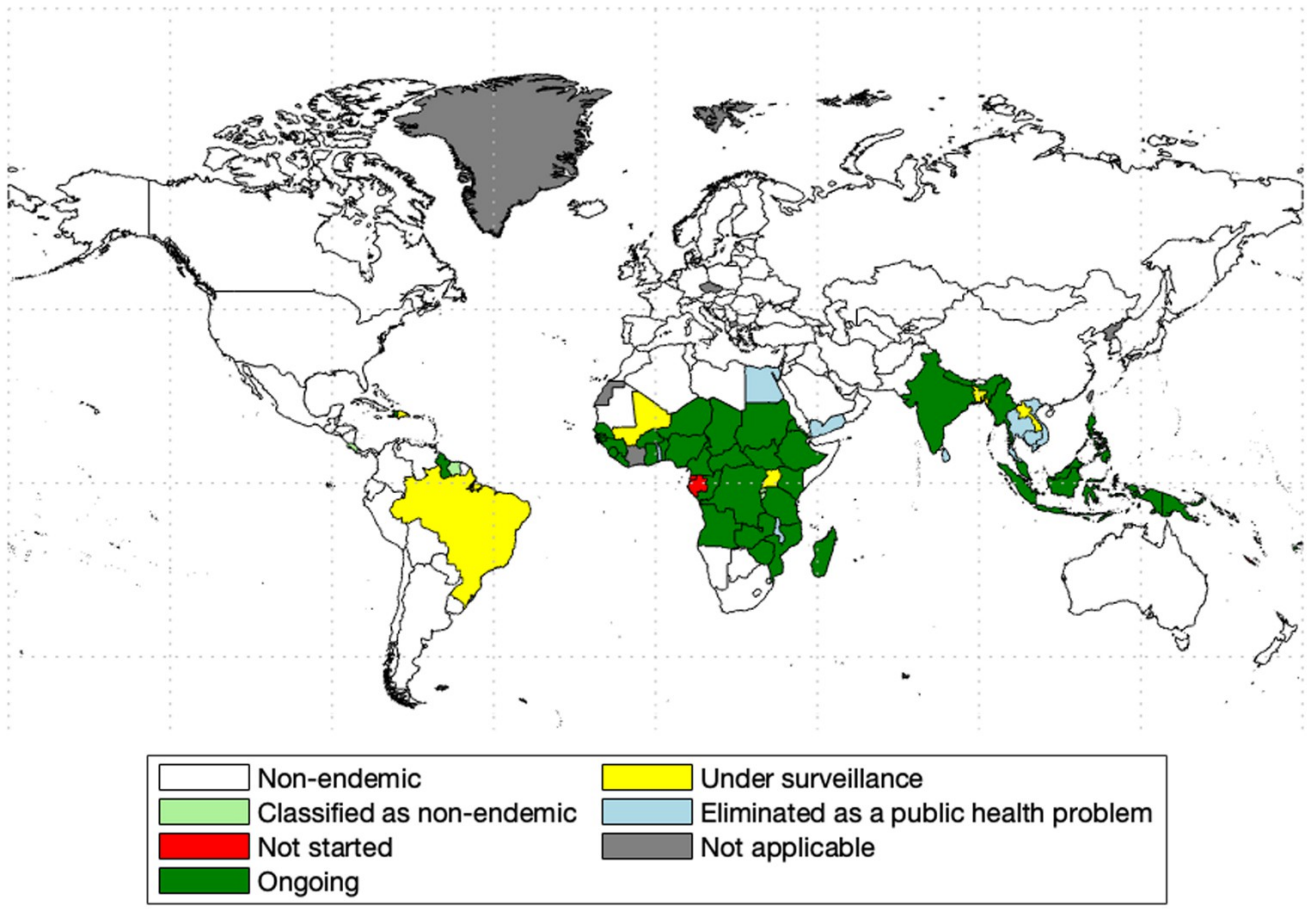
World map of LF and MDA status in 2020. Data collected from [7] and map was made with the aid of borders.m file [8] in MATLAB.

In 2000, WHO launched its Global Programme to Eliminate Lymphatic Filariasis (GPELF) as a public health problem [3]. The primary strategy for LF control and elimination is the WHO recommended preventive chemotherapy [4]. The entire population at risk is treated by mass drug administration (MDA) for at least five consecutive years. In 2020, 863 million people in 50 countries were living in areas that require MDA [3]; see Fig 1. At the same time, GPELF set new goals for the new NTD Road Map (2021-2030) that include reduction to 0 of the total population requiring MDA and 100% of endemic countries implement post-MDA or post-validation surveillance [3]. MDA has already ended and was successful in Dominican Republic [5] but it was not so successful in Haiti [4] and American Samoa [6]. It is therefore important to plan ahead and estimate what can happen at the end of MDA.

Mathematical modeling is a standard and indispensable tool for NTDs elimination efforts [9, 10]. The main mathematical models of LF transmission and control are LYMFASIM [11], EPIFIL [12, 13] and TRANSFIL [14]. The models and their implications for the LF control and elimination through MDA are discussed in [15, 16] or [17]. Furthermore, [18] and [19] created an SI-SI model to investigate the long-term effects of targeted medical treatment in Indonesia. [20] developed an SEI-SI model which was extended by [21] to include possible vaccination and chemoprophylaxis. [22] developed model with vaccination. [23] constructed an SEIQ-SI LF model with quarantine and treatment as control strategies. Also, [24] modeled LF-tuberculosis coinfections and [25] considered global stability and backward bifurcation of their LF transmission model. The cost-effectiveness of different intervention strategies is considered in [26].

In our paper, we adapt a SEI-SI compartmental model by [27] which investigated the effect of MDA on LF transmission in the Philippines. Unlike previous LF modeling papers, we focus our attention on what happens when MDA is terminated and no longer in place. We are interested to see whether the LF transmission can be substantially interrupted by voluntary use of personal protection strategies such as using insect repellents. The research is inspired by [28] and [29] who showed that a voluntary use of DEET can help eliminate dengue or zika virus infections.

We apply the game-theoretic framework developed in [30] and subsequently applied to many diseases, including COVID-19 [31]; see [32] for a recent review. The framework is useful in instances when individuals choose to protect against the mosquito bites and consequently the disease on their own rather than when there are centralized efforts directed towards disease elimination or mosquito control [33]. It has been long established that individuals act in a way that maximizes their self-interests, rather than the interests of the entire group [34]. Voluntary disease protection is prone to free-riding because it produces public goods (reduction of disease prevalence) that have the following two main characteristics [35]: non-rivalry (consumption of a good by one person does not affect the quantities consumed by other individual) and non-exclusion of consumption (impossible to restrict the benefits to certain individuals). The “free-riders” avoid the costs associated with disease prevention while benefiting from other individuals’ actions [36]. Individuals try to balance the real or perceived costs of disease protection against the costs of the disease [37]. The outcomes of different choices of a specific individual depend on the actions chosen by the rest of the population since the behavior of the rest of the population determines the prevalence of the disease and thus the risk of infection to a focal individual. A solution of this game is a concept of Nash equilibrium, a strategy from which nobody prefers to deviate.

We identify such optimal voluntary protection levels and demonstrate that under such conditions, LF incidence rates become too high. Thus, we conclude that voluntary use alone is not a sufficient tool to keep LF eliminated as a public health concern after the end of MDA.

## 2 Mathematical model

In this section we build a mathematical model for the voluntary use of insect repellents and other personal protection means to prevent LF. We first introduce the compartmental model of LF transmission. Then, we add the game-theoretic component that will allow us to investigate individuals’ optimal decisions on choosing their level of protection. Finally, we will calibrate the model based on data from the literature.

### 2.1 Compartmental model

**Fig 2:**
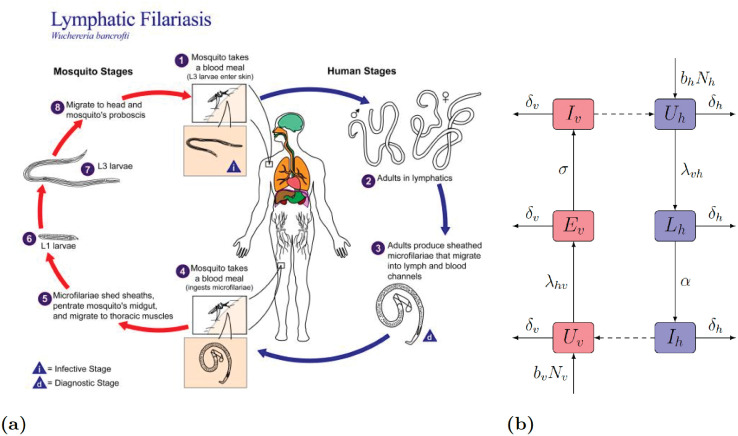
(a) Life cycle of *W. bancrofti*. Image courtesy of Public Health Image Library, Centers for Disease Control and Prevention (https://phil.cdc.gov/Details.aspx?pid=3425). (b) Scheme of the ODE compartmental model for LF transmission from [27] with no treatment (after the termination of MDA). The human population is divided into uninfected *U*_*h*_, latent *L*_*h*_, and infectious *I*_*h*_; the total population is *N*_*h*_ = *U*_*h*_ + *L*_*h*_ + *I*_*h*_. Mosquitoes are either uninfected *U*_*v*_, exposed *E*_*v*_, or infected *I*_*v*_; the total population is *N*_*v*_ = *U*_*v*_ + *E*_*v*_ + *I*_*v*_. Solid arrows represent the transition of humans and mosquitoes between different states of infection. The letters next to the arrows specify the rates of the transitions. All new members of both populations enter their respective uninfected classes at per capita rates *b*_*h*_ and *b*_*v*_. Both humans and mosquitoes leave their respective population through natural death at per capita rates *δ*_*h*_ and *δ*_*v*_. The uninfected mosquitoes become infected at rate $\lambda_{hv}=\beta \theta_{hv}\frac{I_{h}}{N_{h}}$. The uninfected humans become latent at the rate $\lambda_{vh}=\beta \theta_{vh}\frac{I_{v}}{N_{h}}$, the force of infection. The latent individuals progress to infectious at rate *α*. The exposed vectors become infectious at rate *σ*. Dashed lines represent the transfer of parasites from human to mosquito and vice versa through a mosquito bite.

We consider the situation at the hypothetical termination of the MDA treatments. We adapt an ODE compartmental model for LF transmission that was introduced in [27]. Their compartmental model simplified by the absence of MDA but extended by the presence of exposed vectors is shown and described in Fig 2. The parameters are explained in Table 1.

**Table 1:** Model parameters. The rates are per capita per week. The parameter values are discussed in Section 2.3. The range shows the bounds we used in sensitivity and uncertainty analysis in Section 4.1.

Symbol	Description	Value	Range
*b*_*h*_	Human birth rate	6 × 10^−4^	[10^−4^, 10^−3^]
*δ*_*h*_	Human natural death rate	4.2 × 10^−4^	[10^−4^, 10^−3^]
*δ*_*v*_	Mosquito natural death rate	0.1	[0.05, 0.15]
*b*_*v*_	Mosquito birth rate	*δ*_*v*_ + *b*_*h*_ − *δ*_*h*_	
*c*	Proportion of the time the individuals use protection	variable in [0, 1]	
*β*_0_	Maximal mosquito bite rate	1	[0.5, 1.5]
*β*(*c*)	Mosquito bite rate when protecting at *c*	*β*_0_(1 − *c*)	
*θ*_*vh*_	Probability of transmission from mosquito to human	7.5 × 10^−4^	[0, 10^−3^]
*θ*_*hv*_	Probability of transmission from human to mosquito	0.37	[0.2, 0.4]
*α*	Progression rate from *L*_*h*_ to *I*_*h*_	0.0288	[0.02, 0.05]
*σ*	Progression rate from *E*_*v*_ to *I*_*v*_	2/3	[0.1, 1]
*n*_*v*_	Number of mosquitoes per human	3	[0, 5]
*κ*	Cost of maximal protection (relative to cost of LF)	0.1	[0, 1]
*k*(*c*)	Cost of protection (relative to cost of LF) when using *c*	*κc*	

As derived in 3.1, the effective reproduction number is

$$\begin{array}{c} R_{e}=\sqrt{\frac{\beta^{2}\theta_{vh}\theta_{hv}n_{v}\alpha \sigma }{b_{h}b_{v}\left(b_{h}+\alpha \right)\left(b_{v}+\sigma \right)}}. \end{array}$$ (1)

When $R_{e}<1$, then the disease-free equilibrium is locally asymptotically stable and when $R_{e}>1$, then the endemic equilibrium is locally asymptotically stable [38]. Furthermore, if $R_{e}>1$, then the force of infection at the endemic equilibrium is given by

$$\begin{array}{c} \lambda_{vh}^{*}=\beta \theta_{vh}\frac{R_{e}^{2}-1}{\frac{\beta \theta_{vh}}{b_{h}}+R_{e}^{2}\frac{b_{v}+\sigma }{n_{v}\sigma }}. \end{array}$$ (2)

### 2.2 Game-theoretic component

At this point, we add a game-theoretic component to study individual prevention strategies and introduce the following game inspired by the framework introduced in [30].

The players of the game are uninfected individuals who repeatedly chose to protect themselves against mosquito bites. Their strategy is given by a number *c* ∈ [0, 1] that specifies a proportion of the time the individual uses personal protection such as insect repellent to prevent mosquito bites. The strategy *c* influences the mosquito biting rate, *β* = *β*(*c*). For illustrative purposes, we assume *β*(*c*) = *β*_0_(1 − *c*) where *β*_0_ is the maximal mosquito biting rate without any protection. However, our analysis and qualitative results will stay valid for any non-negative decreasing function *β*(*c*) satisfying *β*″(*c*) ≤ 0 on [0, 1].

The protection does not come for free and we assume that to use a strategy *c*, the individual has to pay the cost *k*(*c*). In our examples, we assume *k*(*c*) = *κc* where *κ* is the cost of complete and maximal protection. However, our analysis and qualitative results stay valid for any non-negative increasing function *k*(*c*) satisfying *k*″(*c*) ≤ 0 on [0, 1]. We assume that the cost *k*(*c*) is relative to the cost of the disease, i.e., *k*(*c*) = 1 means that the cost of the protection equals the cost of the disease.

The solution of the game, called the Nash equilibrium, is the population-level value *c*_NE_ at which no individual can increase their own benefits by deviating from the population strategy.

The individual’s benefits, or payoffs, depend on the individual’s strategy but also on the prevalence of LF in the population, i.e., on the strategies of other players. Following [30], we assume that all individuals are provided with the same information such as prevalence of LF in the population, the cost of contracting LF, and the cost of protection. We will also assume that they all use the information in the same and rational way to assess costs and risks.

### 2.3 Model calibration

We adopt most parameter values from [27] and references therein. All rates are expressed per capita per week. We set the human birth rate as *b*_*h*_ = 6 × 10^−4^ and the human death rate as *δ*_*h*_ = 4.2 × 10^−4^ to agree with the population dynamics of the Caraga region, the Philippines. As in [39], we set the mosquito death rate as *δ*_*v*_ = 0.1. In line with [27], to keep the mosquito population to be a constant multiple of *N*_*h*_, we set *b*_*v*_ = *δ*_*v*_ + *b*_*h*_ − *δ*_*h*_. The number of mosquitoes per humans was estimated as *n*_*v*_ = 3. We assume the progression rate from *L*_*h*_ to *I*_*h*_ is *α* = 0.0288 [17]. Also, we assume the maximal mosquito bite rate is *β*_0_ = 1 [39]. The probability of transmission from human to mosquitoes is *θ*_*hv*_ = 0.37 [13]. In vectors, L1 stage larvae needs 1.5 weeks to mature into infectious L3 stage larvae [40], i.e., the rate of progression from *E*_*v*_ to *I*_*v*_ is *σ* = 2/3.

We differ from [27] by setting the probability of transmission from mosquito to human as *θ*_*vh*_ = 7.5 × 10^−4^ = 6.6 × 1.13 × 10^−4^ where 6.6 is the mean saturation level of L3 larvae in mosquitoes [41] and 1.13 × 10^−4^ is the proportion of L3 filarial parasites entering a host which develop into adult worms [13]. We note that [27] used a value *θ*_*vh*_ = 1.13 × 10^−4^, but that gives $R_{e}\approx 1.3$. Our values of *θ*_*vh*_ yields $R_{e}\approx 3.43$. Such a value is more in line with [42] which estimates $R_{e}$ values for LF to be between 2.7 and 30.

Finally, we assume that the cost of (complete) protection, relative to the cost of LF, is given by *κ* = 0.1. We arrived at this estimate as follows. In 2000, a chronic LF patient could lose up to $50 annually due to LF [43]. We adjusted it to $100 annually for today’s value. At the same time, the cost of full protection by DEET was estimated in [29] to about $10.

We investigate the dependence of our result on the parameter values in Section 4.1.

## 3 Analysis

To solve the game, i.e., find the Nash equilibrium and the optimal voluntary protection level, we assume that all players use the same strategy, *c*_pop_, and only the strategy of the focal player, *c*, may vary. We assume that human and mosquito populations are large enough so that the behavior of a single individual does not significantly affect the number of infected mosquitoes.

The effective reproduction number depends on *c*_pop_. Specifically,

$$\begin{array}{c} R_{e}\left(c_{\text{pop}}\right)=\sqrt{\frac{\beta^{2}\left(c_{\text{pop}}\right)\theta_{vh}\theta_{hv}n_{v}\alpha \sigma }{b_{h}b_{v}\left(b_{h}+\alpha \right)\left(b_{v}+\sigma \right)}}. \end{array}$$ (3)

Assuming *β*(*c*_pop_) = *β*_0_(1 − *c*_pop_), we get

$$\begin{array}{c} R_{e}\left(c_{\text{pop}}\right)=\left(1-c_{\text{pop}}\right)R_{e}\left(0\right). \end{array}$$ (4)

When $R_{e}\left(c_{\text{pop}}\right)\leq 1$, the population will reach disease-free equilibrium. When $R_{e}\left(c_{\text{pop}}\right)>1$, i.e., when *c*_pop_ ∈ [0, *c*_max_] where

$$\begin{array}{c} c_{\text{max}}=1-\frac{1}{R_{e}\left(0\right)}, \end{array}$$ (5)

the population will reach the endemic equilibrium. Here, *c*_max_ is the maximal protection level at which $R_{e}\geq 1$ and the disease-free equilibrium is not stable. We will assume $R_{e}\left(0\right)>1$ and *c*_pop_ ∈ [0, *c*_max_] as otherwise the disease is eliminated and thus there is no need for a further analysis. As common in game-theoretical models, we will assume that the population actually is in the endemic equilibrium [30].

An uninfected focal individual in *U*_*h*_ using a strategy *c* when everyone else uses a strategy *c*_pop_ contracts the infection and moves to *L*_*h*_ at rate $\beta \left(c\right)\theta_{vh}\frac{I_{v}}{N_{h}}$. Note that the ratio $i_{v}=\frac{I_{v}}{N_{h}}$ depends on the strategy *c*_pop_, see Eq (47) in Section 3.1. The rate is thus given by

$$\begin{array}{c} \lambda_{vh}\left(c,c_{\text{pop}}\right)=\beta \left(c\right)\theta_{vh}i_{v}^{*}\left(c_{\text{pop}}\right) \end{array}$$ (6)

where

$$\begin{array}{c} i_{v}^{*}\left(c_{\text{pop}}\right)=\frac{R_{e}^{2}\left(c_{\text{pop}}\right)-1}{\frac{\beta \left(c_{\text{pop}}\right)\theta_{vh}}{b_{h}}+R_{e}^{2}\left(c_{\text{pop}}\right)\frac{b_{v}+\sigma }{n_{v}\sigma }}. \end{array}$$ (7)

As in [30], the payoff to the focal individual is the negative expected cost of getting the infection minus the cost of individual protection, i.e.,

$$\begin{array}{c} E\left(c,c_{\text{pop}}\right)=-\frac{\lambda_{vh}\left(c,c_{\text{pop}}\right)}{\lambda_{vh}\left(c,c_{\text{pop}}\right)+\delta_{h}}-k\left(c\right), \end{array}$$ (8)

where λ_*vh*_/(λ_*vh*_ + *δ*_*h*_) is the probability that an uninfected individual contracts the infection.

To solve for the Nash equilibrium, we need to find a protection level *c*_NE_ such that the function *f*(*c*) = *E*(*c*, *c*_NE_) on [0, 1], attains its maximum at *c* = *c*_NE_. We note that while the population strategy *c*_NE_ must be between 0 and *c*_max_, the individual strategy can still be between 0 (no protection) and 1 (complete protection). We have

$$\frac{\partial }{\partial c}E\left(c,c_{\text{pop}}\right)=-\frac{\delta_{h}\cdot \frac{\partial }{\partial c}\lambda_{vh}\left(c,c_{\text{pop}}\right)}{{\left(\lambda_{vh}\left(c,c_{\text{pop}}\right)+\delta_{h}\right)}^{2}}-k^{\prime }\left(c\right),$$ (9)

$$\frac{\partial^{2}}{\partial c^{2}}E\left(c,c_{\text{pop}}\right)=2\frac{\delta_{h}\cdot {\left(\frac{\partial }{\partial c}\lambda_{vh}\left(c,c_{\text{pop}}\right)\right)}^{2}}{{\left(\lambda_{vh}\left(c,c_{\text{pop}}\right)+\delta_{h}\right)}^{3}}-\frac{\frac{\partial^{2}}{\partial c^{2}}\lambda_{vh}\left(c,c_{\text{pop}}\right)}{{\left(\lambda_{vh}\left(c,c_{\text{pop}}\right)+\delta_{h}\right)}^{2}}-k^{\prime \prime }\left(c\right).$$ (10)

Because *k*″(*c*) ≤ 0 and $\frac{\partial^{2}}{\partial c^{2}}\lambda_{vh}\left(c,c_{\text{pop}}\right)=\beta^{\prime \prime }\left(c\right)\frac{\lambda_{vh}\left(c_{\text{pop}},c_{\text{pop}}\right)}{\beta (c_{\text{pop}})}\leq 0$, it follows that $\frac{\partial^{2}}{\partial c^{2}}E\left(c,c_{\text{pop}}\right)>0$.

Thus, the function *c* → *E*(*c*, *c*_pop_) attains its maximum either at *c* = 0 or *c* = 1. Thus, the Nash equilibrium can be only *c*_NE_ = 0, *c*_NE_ = 1, or a solution of *E*(0, *c*_NE_) = *E*(1, *c*_NE_). Considering the last option, we get, by (8) and (6), at Nash equilibrium,

$$\begin{array}{c} i_{v,NE}^{*}=(\frac{\delta_{h}}{\beta_{0}\theta_{vh}})(\frac{\kappa }{1-\kappa }). \end{array}$$ (11)

Thus, by (7), *c*_NE_ is a solution of

$$\begin{array}{c} 0={\left(1-c\right)}^{2}R_{e}^{2}\left(0\right)(1-\frac{b_{v}+\sigma }{n_{v}\sigma }i_{v,NE}^{*})-\left(1-c\right)\frac{\beta_{0}\theta_{vh}}{b_{h}}i_{v,NE}^{*}-1. \end{array}$$ (12)

### 3.1 Detailed calculations of steady states

The compartmental model in Fig 2 yields the following system of differential equations.

$$\frac{dU_{h}}{dt}=b_{h}N_{h}-(\delta_{h}+\beta \theta_{vh}\frac{I_{v}}{N_{h}})U_{h}$$ (13)

$$\frac{dL_{h}}{dt}=\beta \theta_{vh}\frac{I_{v}}{N_{h}}U_{h}-\left(\delta_{h}+\alpha \right)L_{h}$$ (14)

$$\frac{dI_{h}}{dt}=\alpha L_{h}-\delta_{h}I_{h}$$ (15)

$$\frac{dU_{v}}{dt}=b_{v}N_{v}-(\delta_{v}+\beta \theta_{hv}\frac{I_{h}}{N_{h}})U_{v}$$ (16)

$$\frac{dE_{v}}{dt}=\beta \theta_{hv}\frac{I_{h}}{N_{h}}U_{v}-\left(\delta_{v}+\sigma \right)E_{v}$$ (17)

$$\frac{dI_{v}}{dt}=\sigma E_{v}-\delta_{v}I_{v}.$$ (18)

We set $u_{h}=\frac{U_{h}}{N_{h}}$, $l_{h}=\frac{L_{h}}{N_{h}}$, $i_{h}=\frac{I_{h}}{N_{h}}$, $u_{v}=\frac{U_{v}}{N_{h}}$, $e_{v}=\frac{E_{v}}{N_{h}}$, and $i_{v}=\frac{I_{v}}{N_{h}}$. Using *b*_*v*_ = *δ*_*v*_ + *b*_*h*_ − *δ*_*h*_, this yields,

$$\frac{du_{h}}{dt}=b_{h}-(b_{h}+\beta \theta_{vh}i_{v})u_{h}$$ (19)

$$\frac{dl_{h}}{dt}=\beta \theta_{vh}i_{v}u_{h}-\left(b_{h}+\alpha \right)l_{h}$$ (20)

$$\frac{di_{h}}{dt}=\alpha l_{h}-b_{h}i_{h}$$ (21)

$$\frac{du_{v}}{dt}=b_{v}n_{v}-(b_{v}+\beta \theta_{hv}i_{h})u_{v}$$ (22)

$$\frac{de_{v}}{dt}=\beta \theta_{hv}i_{h}u_{v}-\left(b_{v}+\sigma \right)e_{v}$$ (23)

$$\frac{di_{v}}{dt}=\sigma e_{v}-b_{v}i_{v}.$$ (24)

The steady states are thus given as solution of the following system of algebraic equations.

$$0=b_{h}-(b_{h}+\beta \theta_{vh}i_{v})u_{h}$$ (25)

$$0=\beta \theta_{vh}i_{v}u_{h}-\left(b_{h}+\alpha \right)l_{h}$$ (26)

$$0=\alpha l_{h}-b_{h}i_{h}$$ (27)

$$0=b_{v}n_{v}-(b_{v}+\beta \theta_{hv}i_{h})u_{v}$$ (28)

$$0=\beta \theta_{hv}i_{h}u_{v}-\left(b_{v}+\sigma \right)e_{v}$$ (29)

$$0=\sigma e_{v}-b_{v}i_{v}.$$ (30)

There are two sets of solutions of (25)–(30). The disease-free equilibrium $E^{0}=\left(u_{h}^{0},l_{h}^{0},i_{h}^{0},u_{v}^{0},e_{v}^{0},i_{v}^{0}\right)$ is given by

$$\begin{array}{c} E^{0}=(1,0,0,n_{v},0,0). \end{array}$$ (31)

The effective reproduction number can be derived using the next-generation matrix method [38], or directly as follows. The infected vector stays infected for the time $b_{v}^{-1}$. During that time, it infects individuals at rate *βθ*_*vh*_. The latently infected individuals become infectious with probability $\frac{\alpha }{b_{h}+\alpha }$. Infectious individuals stay infectious for time $b_{h}^{-1}$ and they infect vectors at rate *βθ*_*hv*_*n*_*v*_. The exposed vectors become infectious with probability $\frac{\sigma }{b_{v}+\sigma }$. Thus,

$$\begin{array}{c} R_{e}=\sqrt{\frac{\beta^{2}\theta_{vh}\theta_{hv}n_{v}\alpha \sigma }{b_{h}b_{v}\left(b_{h}+\alpha \right)\left(b_{v}+\sigma \right)}}. \end{array}$$ (32)

We solve for the endemic equilibrium $E^{*}=\left(u_{h}^{*},l_{h}^{*},i_{h}^{*},u_{v}^{*},e_{v}^{*},i_{v}^{*}\right)$, we do the following. By (25),

$$u_{h}^{*}=\frac{1}{1+\frac{\beta \theta_{vh}}{b_{h}}i_{v}^{*}}$$ (33)

$$l_{h}^{*}=\frac{\beta \theta_{vh}}{b_{h}+\alpha }i_{v}^{*}u_{h}^{*}$$ (34)

$$i_{h}^{*}=\frac{\alpha }{b_{h}}l_{h}^{*}$$ (35)

$$u_{v}^{*}=\frac{n_{v}}{1+\frac{\beta \theta_{hv}}{b_{v}}i_{h}^{*}}$$ (36)

$$e_{v}^{*}=\frac{\beta \theta_{hv}}{b_{v}+\sigma }i_{h}^{*}u_{v}^{*}$$ (37)

$$i_{v}^{*}=\frac{\sigma }{b_{v}}e_{v}^{*}.$$ (38)

Thus, by sequentially plugging (33)–(37) into (38), we get

$$i_{v}^{*}=\frac{\sigma }{b_{v}}e_{v}^{*}$$ (39)

$$=\frac{\sigma }{b_{v}}\frac{\beta \theta_{hv}}{b_{v}+\sigma }i_{h}^{*}u_{v}^{*}$$ (40)

$$=\frac{\sigma }{b_{v}}\frac{\beta \theta_{hv}}{b_{v}+\sigma }\frac{\alpha }{b_{h}}l_{h}^{*}\frac{n_{v}}{1+\frac{\beta \theta_{hv}}{b_{v}}i_{h}^{*}}$$ (41)

$$=\frac{\sigma }{b_{v}}\frac{\beta \theta_{hv}}{b_{v}+\sigma }\frac{\alpha }{b_{h}}\frac{\beta \theta_{vh}}{b_{h}+\alpha }i_{v}^{*}u_{h}^{*}\frac{n_{v}}{1+\frac{\beta \theta_{hv}}{b_{v}}\frac{\alpha }{b_{h}}l_{h}^{*}}$$ (42)

$$=\frac{\sigma }{b_{v}}\frac{\beta \theta_{hv}}{b_{v}+\sigma }\frac{\alpha }{b_{h}}\frac{\beta \theta_{vh}}{b_{h}+\alpha }i_{v}^{*}\frac{1}{1+\frac{\beta \theta_{vh}}{b_{h}}i_{v}^{*}}\frac{n_{v}}{1+\frac{\beta \theta_{hv}}{b_{v}}\frac{\alpha }{b_{h}}\frac{\beta \theta_{vh}}{b_{h}+\alpha }i_{v}^{*}u_{h}^{*}}$$ (43)

$$=\frac{\sigma }{b_{v}}\frac{\beta \theta_{hv}}{b_{v}+\sigma }\frac{\alpha }{b_{h}}\frac{\beta \theta_{vh}}{b_{h}+\alpha }i_{v}^{*}\frac{1}{1+\frac{\beta \theta_{vh}}{b_{h}}i_{v}^{*}}\frac{n_{v}}{1+\frac{\beta \theta_{hv}}{b_{v}}\frac{\alpha }{b_{h}}\frac{\beta \theta_{vh}}{b_{h}+\alpha }i_{v}^{*}\frac{1}{1+\frac{\beta \theta_{vh}}{b_{h}}i_{v}^{*}}}$$ (44)

$$=R_{e}^{2}\frac{1}{1+\frac{\beta \theta_{vh}}{b_{h}}i_{v}^{*}+\frac{\beta \theta_{hv}}{b_{v}}\frac{\alpha }{b_{h}}\frac{\beta \theta_{vh}}{b_{h}+\alpha }i_{v}^{*}}i_{v}^{*}$$ (45)

$$=R_{e}^{2}\frac{1}{1+\left(\frac{\beta \theta_{vh}}{b_{h}}+R_{e}^{2}\frac{b_{v}+\sigma }{n_{v}\sigma }\right)i_{v}^{*}}i_{v}^{*}.$$ (46)

Hence, either $i_{v}^{*}=0$, or

$$\begin{array}{c} i_{v}^{*}=\frac{R_{e}^{2}-1}{\frac{\beta \theta_{vh}}{b_{h}}+R_{e}^{2}\frac{b_{v}+\sigma }{n_{v}\sigma }}. \end{array}$$ (47)

It follows that the endemic equilibrium exists only if $R_{e}>1$. Once $i_{v}^{*}$ is evaluated by (47), the formulas (33)–(37) then yield values of the remaining compartments in the endemic equilibrium.

Furthermore,

$$\begin{array}{c} \lambda_{vh}^{*}=\beta \theta_{vh}i_{v}^{*}=\beta \theta_{vh}\frac{R_{e}^{2}-1}{\frac{\beta \theta_{vh}}{b_{h}}+R_{e}^{2}\frac{b_{v}+\sigma }{n_{v}\sigma }}. \end{array}$$ (48)

## 4 Results

For the parameter values specified in Table 1, the population level protection leading to elimination of LF is given by *c*_max_ ≈ 0.71 while the optimal voluntary protection level is *c*_NE_ ≈ 0.70. The annual incidence rate when individuals use the optimal voluntary level of protection is about 112 cases per 10^5^ individuals. We can thus see that after the termination of MDA, the disease would not be eliminated as a public health concern by optimal voluntary use of personal protection alone.

**Fig 3:**
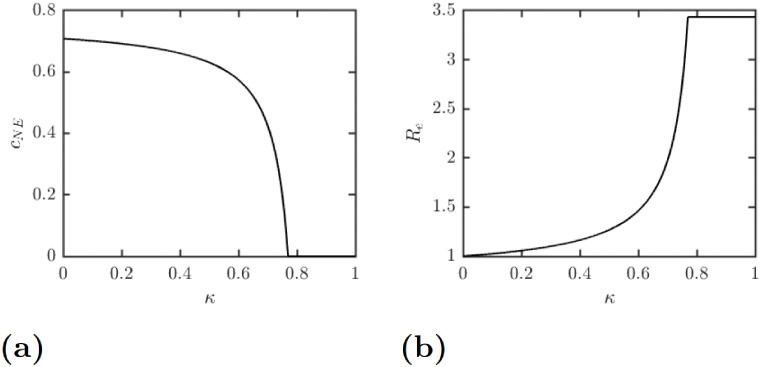
The dependence of (a) the optimal individual protection levels *c*_NE_ and (b) the effective reproduction number $R_{e}$ on the relative cost of protection the full protection, *κ*.

Fig 3a shows the dependence of the optimal individual protection levels *c*_NE_ on the relative cost of protection the full protection, *κ*. Once the cost of protection grows above 0.77, *c*_NE_ = 0. It means that if the cost of protection is higher than about 3/4 of the cost of LF, it is not beneficial to use any personal protection at all. On the other hand, when the cost of protection is very low, *c*_NE_ ≈ *c*_max_, meaning that LF would be very close to elimination.

Similarly, Fig 3b shows the dependence of the effective reproduction number on *κ*. In agreement with Fig 3a, when *κ* ≈ 0, $R_{e}\approx 1$ and when *κ* > 3/4, $R_{e}\approx 3.43$. Note that as long as *κ* > 0, $R_{e}>1$, i.e., the optimal voluntary use of protection will never completely eliminate the disease on its own.

### 4.1 Uncertainty and sensitivity analysis

We performed uncertainty and sensitivity analysis using the Latin hyper-cube sampling with partial rank correlation coefficient (LHS-PRCC) scheme [44, 45]. The scheme is described in detail in [46] and the MATLAB and R implementation can be found in [47].

**Fig 4:**
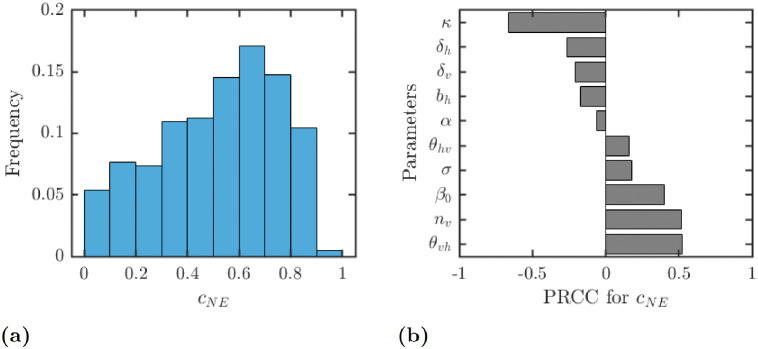
Results of the uncertainty (a) and sensitivity (b) analysis for the dependence of *c*_NE_ on parameter values. The parameter ranges are as in Table 1. Only parameters with sensitivity over 0.05 are shown in figure (b).

Fig 4a shows the results of uncertainty analysis, i.e., the distribution of *c*_NE_ among all the sampled parameter values. The most frequent value of *c*_NE_ is around 0.75 with the average value of above 0.53.

Fig 4b shows the sensitivity of *c*_NE_ on various parameters. There is a strong negative correlation between the optimal voluntary protection level *c*_NE_ and the cost of protection, *κ*. Increasing *κ* decreases *c*_NE_. The human or mosquito death rates or the human birth rate also has a negative effect on *c*_NE_. On the other hand, there is a positive correlation between *c*_NE_ and the probability of transmission from vector to humans, *θ*_*vh*_, the number of mosquitoes per human, *n*_*v*_, and the maximal transmission rate *β*_0_. Increasing any of these parameters will increase *c*_NE_. The correlations between *c*_NE_ and the probability of transmission from human to vectors, *θ*_*hv*_ or the incubation rate *σ* are positive but relatively small. The correlation with the progression rate from *L*_*h*_ to *I*_*h*_, *α*, is negligible.

**Fig 5:**
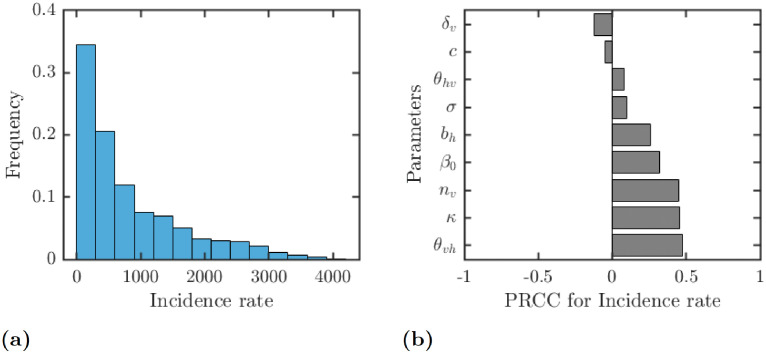
Results of the uncertainty (a) and sensitivity (b) analysis for the dependence of the annual incidence rate (per 10^5^ individuals) on various parameters. The parameter ranges are as in Table 1. Only parameters with sensitivity over 0.05 are shown in figure (b).

We note that the actual value of *c*_NE_ is not as important as the annual incidence rate of LF when everybody adopts the optimal voluntary strategy. As seen from Fig 5a, the incidence rate is typically quite large which demonstrates that our results are robust and not overly affected by parameter changes. As shown in Fig 5b, the incidence rate is positively correlated with *κ*, *θ*_*vh*_, *n*_*v*_ as well as with *β*_0_ and *b*_*h*_. The incidence rate is negatively correlated with the vector death rate *δ*_*v*_.

**Fig 6:**
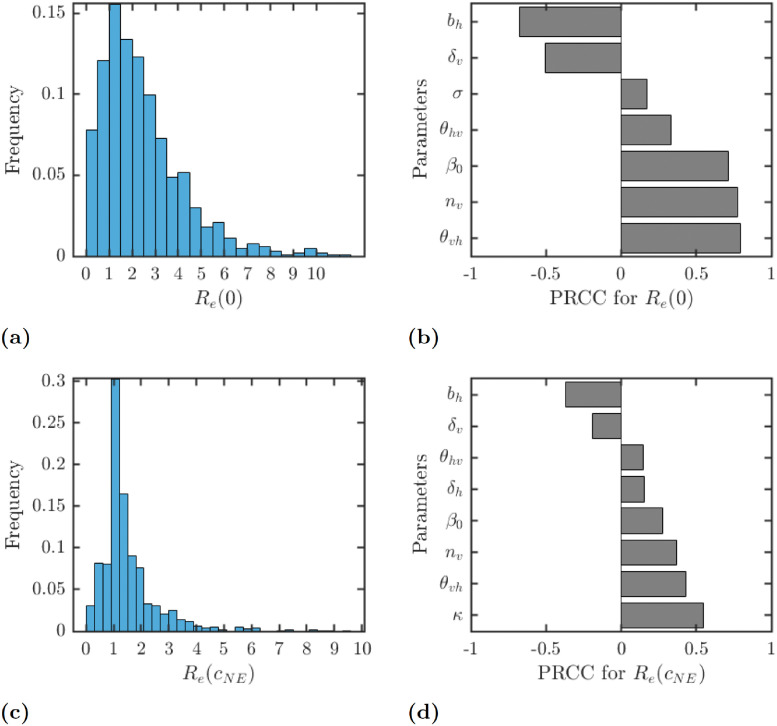
Results of the uncertainty (a) and (c), and sensitivity (b) and (d) analysis for the dependence of $R_{e}\left(0\right)$ and $R_{e}\left(c_{\text{NE}}\right)$ on various parameters. The parameter ranges are as in Table 1. Only parameters with sensitivity over 0.05 are shown in figures (b) and (d).

Finally, we investigate the sensitivity of $R_{e}\left(0\right)$ and $R_{e}\left(c_{\text{NE}}\right)$ on the parameters. It follows directly from formula (1) and it is also illustrated in Fig 6 that $R_{e}\left(0\right)$ positively correlates with *θ*_*vh*_, *n*_*v*_ and *β*_0_ and negatively with *b*_*h*_ and *δ*_*v*_. The sensitivity of $R_{e}\left(c_{\text{NE}}\right)$ is similar; moreover, $R_{e}\left(c_{\text{NE}}\right)$ is most sensitive on *κ*. We note that the average value of $R_{e}\left(0\right)$ is approximately 2.45 and the average value of $R_{e}\left(c_{\text{NE}}\right)$ is approximately 1.53. The latter fact again indicates that voluntary prevention of LF will not significantly help with elimination efforts.

## 5 Conclusions and discussion

We applied the game-theoretic framework [30] to the compartmental model of LF transmission [27]. We identified optimal voluntary protection levels against mosquito bites and estimated the annual incidence rate in a hypothetical scenario when the whole population uses this level of protection. We demonstrated that the LF incidence rates remain too high. Thus, we can conclude that the voluntary use of insect repellents alone is not sufficient to keep LF eliminated as a public health concern after the end of MDA.

Our result underlines the critical importance of conducting the Transmission Assessment Surveys (TAS) to properly define endpoints MDA [48].

We calibrated our model based on the data from literature and performed uncertainty and sensitivity analysis to understand how different parameter values influence the outcomes. However, there is an ongoing need to strengthen data collection and evaluation for decision-making [49].

Unlike previous models of LF transmission that focused on disease control and treatment on the population level, our model focuses on voluntary individual use of prevention.

On one hand, our main finding that voluntary prevention alone is not enough to eliminate LF is not surprising. Similar results have been already demonstrated in a general scenario [50] as well as for specific diseases such as typhoid fever [51], polio [52], cholera [53] or Hepatitis B [54, 55]. In all cases, the results are caused by a high cost of prevention relative to the cost of the disease.

On the other hand, our results is in striking contrast with models for other vector-borne diseases such as malaria [56], dengue [28], chikungunya [57] and visceral leishmaniasis [58] or diseases like Ebola [59]. It should be noted that in all these cases, cost of disease prevention is low relative to the cost of the disease.

Our model can be further improved in several ways. We assumed that individuals have perfect information about the LF epidemics and the protection coverage in the population. This is almost certainly not the case. In fact, the knowledge about LF and its transmission can be quite low [60]. This means that the perceived risk of LF and subsequently the optimal voluntary protection levels will be lower than predicted by our model. This will, in turn, cause the incidence rates to be even higher. Furthermore, we assumed that individuals are rational and base their decision solely on the expected payoff. However, individuals have different risk perceptions [61] and also base their decision on different social aspects [62]. Therefore, many recent studies now use multi-agent-simulation (MAS) methodology which allows more flexibility and realism [63–69]. Despite these shortcomings, the general framework used in our model still works well and has been shown to predict incidence rate of Chagas disease based on the cost of protection (insecticide-treated nests) in various countries [70].

The above mathematical models in aggregate show a potential path towards NTDs elimination by leveraging individual’s decisions and interests. The key is to increase individuals’ knowledge about the diseases in general. While the cost of insect repellents alone may be too large to offset the risk of LF, avoiding mosquito bites also prevents the risk of other vector-borne diseases. This lowers the relative cost of protection and makes the bite prevention a rational choice. Thus, a coordinated educational campaign aimed at all common mosquito transmitted diseases may be a low cost tool with large benefits that should be used in disease elimination efforts.

## References

[1] Chandy A, Thakur AS, Singh MP, Manigauha A. A review of neglected tropical diseases: filariasis. Asian Pacific Journal of Tropical Medicine. 2011;4(7):581–586. doi: 10.1016/S1995-7645(11)60150-8 21803313

[2] Mathew CG, Bettis AA, Chu BK, English M, Ottesen EA, Bradley MH, et al. The health and economic burdens of lymphatic filariasis prior to mass drug administration programs. Clinical Infectious Diseases. 2020;70(12):2561–2567. doi: 10.1093/cid/ciz671 31343064 PMC7286370

[3] WHO. World Health Organization: Lymphatic filariasis; 2022. https://www.who.int/news-room/fact-sheets/detail/lymphatic-filariasis.

[4] Hast MA, Javel A, Denis E, Barbre K, Rigodon J, Robinson K, et al. Positive-case follow up for lymphatic filariasis after a transmission assessment survey in Haiti. PLoS Neglected Tropical Diseases. 2022;16(2):e0010231. doi: 10.1371/journal.pntd.0010231 35213537 PMC8906642

[5] Gonzales M, Noland GS, Mariano EF, Blount S. Lymphatic filariasis elimination in the Dominican Republic: History, progress, and remaining steps. PLoS Neglected Tropical Diseases. 2021;15(8):e0009590. doi: 10.1371/journal.pntd.0009590 34375332 PMC8378723

[6] Sheel M, Sheridan S, Gass K, Won K, Fuimaono S, Kirk M, et al. Identifying residual transmission of lymphatic filariasis after mass drug administration: Comparing school-based versus community-based surveillance-American Samoa, 2016. PLoS Neglected Tropical Diseases. 2018;12(7):e0006583. doi: 10.1371/journal.pntd.0006583 30011276 PMC6062125

[7] WHO. Lymphatic filariasis. Status of Mass Drug Administration: 2020; 2022. https://apps.who.int/neglected_diseases/ntddata/lf/lf.html.

[8] Greene CA, Thirumalai K, Kearney KA, Delgado JM, Schwanghart W, Wolfenbarger NS, et al. The Climate Data Toolbox for MATLAB. Geochemistry, Geophysics, Geosystems. 2019;. doi: 10.1029/2019GC008392

[9] Anderson RM, May RM. Infectious diseases of humans: dynamics and control. Oxford University Press; 1992.

[10] Behrend MR, Basáñez MG, Hamley JI, Porco TC, Stolk WA, Walker M, et al. Modelling for policy: the five principles of the Neglected Tropical Diseases Modelling Consortium. PLoS Neglected Tropical Diseases. 2020;14(4):e0008033. doi: 10.1371/journal.pntd.0008033 32271755 PMC7144973

[11] Stolk WA, De Vlas SJ, Borsboom GJ, Habbema JDF. LYMFASIM, a simulation model for predicting the impact of lymphatic filariasis control: quantification for African villages. Parasitology. 2008;135(13):1583–1598. doi: 10.1017/S0031182008000437 19006602

[12] Chan MS, Srividya A, Norman R, Pani S, Ramaiah KD, Vanamail P, et al. Epifil: a dynamic model of infection and disease in lymphatic filariasis. American Journal of Tropical Medicine and Hygiene. 1998;59(4):606–614. doi: 10.4269/ajtmh.1998.59.606 9790439

[13] Norman R, Chan MS, Srividya A, Pani S, Ramaiah KD, Vanamail P, et al. EPIFIL: the development of an age-structured model for describing the transmission dynamics and control of lymphatic filariasis. Epidemiology & Infection. 2000;124(3):529–541. doi: 10.1017/s0950268899003702 10982078 PMC2810940

[14] Irvine MA, Reimer LJ, Njenga SM, Gunawardena S, Kelly-Hope L, Bockarie M, et al. Modelling strategies to break transmission of lymphatic filariasis-aggregation, adherence and vector competence greatly alter elimination. Parasites & Vectors. 2015;8(1):1–19. doi: 10.1186/s13071-015-1152-3 26489753 PMC4618540

[15] Michael E, Malecela-Lazaro MN, Kabali C, Snow LC, Kazura JW. Mathematical models and lymphatic filariasis control: endpoints and optimal interventions. Trends in Parasitology. 2006;22(5):226–233. doi: 10.1016/j.pt.2006.03.005 16564745

[16] Stone CM, Kastner R, Steinmann P, Chitnis N, Tanner M, Tediosi F. Modelling the health impact and cost-effectiveness of lymphatic filariasis eradication under varying levels of mass drug administration scale-up and geographic coverage. BMJ Global Health. 2016;1(1):e000021. doi: 10.1136/bmjgh-2015-000021 28588916 PMC5321305

[17] Jambulingam P, Subramanian S, De Vlas S, Vinubala C, Stolk W. Mathematical modelling of lymphatic filariasis elimination programmes in India: required duration of mass drug administration and post-treatment level of infection indicators. Parasites & Vectors. 2016;9(1):1–18. doi: 10.1186/s13071-016-1768-y 27624157 PMC5022201

[18] Supriatna A, Serviana H, Soewono E. A mathematical model to investigate the long-term effects of the lymphatic filariasis medical treatment in Jati Sampurna, West Java. Inst Tech Bandung J Sci. 2009;41(1):1–14.

[19] Supriatna AK, Anggriani N. Lymphatic filariasis transmission and control: a mathematical modelling approach. In: Alfonso JR-M, ed Book chapter in Current Tropics in Tropical Medicine. 2012; p. 425–442.

[20] Bhunu C, Mushayabasa S. Transmission dynamics of lymphatic filariasis: a mathematical approach. International Scholarly Research Network, ISRN Biomathematics, Volume 2012, Article ID 930130, 9 pages doi: 10.5402/2012/930130 https://downloads.hindawi.com/archive/2012/930130.pdf.

[21] Bhunu CP. Assessing the potential of pre-exposure vaccination and chemoprophylaxis in the control of lymphatic filariasis. Applied Mathematics and Computation. 2015;250:571–579. doi: 10.1016/j.amc.2014.11.018

[22] Simelane S, Mwamtobe P, Abelman S, Tchuenche J. A Mathematical Model for the Transmission Dynamics of Lymphatic Filariasis with Intervention Strategies. Acta Biotheoretica. 2020;68(3):297–320. doi: 10.1007/s10441-019-09370-y 31758278

[23] Mwamtobe PM, Simelane SM, Abelman S, Tchuenche JM. Mathematical analysis of a lymphatic filariasis model with quarantine and treatment. BMC Public Health. 2017;17(1):1–13. doi: 10.1186/s12889-017-4160-8 28302096 PMC5356380

[24] Iyare EB, Akhaze RU, Ako II. Mathematical Analysis of A Tuberculosis-Lymphatic filariasis Co-infection Model. ResearchSquare. 2021;.

[25] Iyare EB, Okuonghae D, Osagiede F. Global Stability and Backward Bifurcation for a Lymphatic filariasis model. ResearchSquare. 2021;.

[26] Darmawati D, Musafira M, Ekawati D, Nur W, Muhlis M, Azzahra SF. Sensitivity, Optimal Control, and Cost-Effectiveness Analysis of Intervention Strategies of Filariasis. Jambura Journal of Mathematics. 2022;4(1):64–76. doi: 10.34312/jjom.v4i1.11766

[27] Salonga PKN, Mendoza VMP, Mendoza RG, Belizario VY Jr. A mathematical model of the dynamics of lymphatic filariasis in Caraga Region, the Philippines. Royal Society Open Science. 2021;8(6):201965. doi: 10.1098/rsos.201965 34234950 PMC8242838

[28] Dorsett C, Oh H, Paulemond ML, Rychtář J. Optimal repellent usage to combat dengue fever. Bulletin of Mathematical Biology. 2016;78(5):916–922. doi: 10.1007/s11538-016-0167-z 27142427

[29] Angina J, Bachhu A, Talati E, Talati R, Rychtář J, Taylor D. Game-theoretical model of the voluntary use of insect repellents to prevent Zika fever. Dynamic Games and Applications. 2022;12:133–146. doi: 10.1007/s13235-021-00418-8 35127230 PMC8800840

[30] Bauch CT, Earn DJ. Vaccination and the theory of games. Proceedings of the National Academy of Sciences. 2004;101(36):13391–13394. doi: 10.1073/pnas.0403823101 15329411 PMC516577

[31] Agusto FB, Erovenko IV, Fulk A, Abu-Saymeh Q, Romero-Alvarez D, Ponce J, et al. To isolate or not to isolate: The impact of changing behavior on COVID-19 transmission. BMC Public Health. 2022;22(1):1–20. doi: 10.1186/s12889-021-12275-6 35057770 PMC8771191

[32] Chang SL, Piraveenan M, Pattison P, Prokopenko M. Game theoretic modelling of infectious disease dynamics and intervention methods: a review. Journal of Biological Dynamics. 2020;14(1):57–89. doi: 10.1080/17513758.2020.1720322 31996099

[33] Funk S, Salathé M, Jansen VA. Modelling the influence of human behaviour on the spread of infectious diseases: a review. Journal of the Royal Society Interface. 2010;7(50):1247–1256. doi: 10.1098/rsif.2010.0142 20504800 PMC2894894

[34] Maskin E. Nash equilibrium and welfare optimality. The Review of Economic Studies. 1999;66(1):23–38. doi: 10.1111/1467-937X.00076

[35] Ibuka Y, Li M, Vietri J, Chapman GB, Galvani AP. Free-riding behavior in vaccination decisions: an experimental study. PloS one. 2014;9(1). doi: 10.1371/journal.pone.0087164 24475246 PMC3901764

[36] Serpell L, Green J. Parental decision-making in childhood vaccination. Vaccine. 2006;24(19):4041–4046. doi: 10.1016/j.vaccine.2006.02.037 16530892

[37] Neilan RLM, Schaefer E, Gaff H, Fister KR, Lenhart S. Modeling optimal intervention strategies for Cholera. Bulletin of Mathematical Biology. 2010;72(8):2004–2018. doi: 10.1007/s11538-010-9521-8 20204710

[38] van den Driessche P, Watmough J. Reproduction numbers and sub-threshold endemic equilibria for compartmental models of disease transmission. Mathematical Biosciences. 2002;180:29–48. doi: 10.1016/S0025-5564(02)00108-6 12387915

[39] de los Reyes AA, Escaner JML. Dengue in the Philippines: model and analysis of parameters affecting transmission. Journal of Biological Dynamics. 2018;12(1):894–912. doi: 10.1080/17513758.2018.1535096 30353774

[40] Paily K, Hoti S, Das P. A review of the complexity of biology of lymphatic filarial parasites. Journal of Parasitic Diseases. 2009;33(1):3–12. doi: 10.1007/s12639-009-0005-4 23129882 PMC3454129

[41] Subramanian S, Krishnamoorthy K, Ramaiah K, Habbema J, Das P, Plaisier A. The relationship between microfilarial load in the human host and uptake and development of *Wuchereria bancrofti* microfilariae by *Culex quinquefasciatus*: a study under natural conditions. Parasitology. 1998;116(3):243–255. doi: 10.1017/S0031182097002254 9550218

[42] Moraga P, Cano J, Baggaley RF, Gyapong JO, Njenga SM, Nikolay B, et al. Modelling the distribution and transmission intensity of lymphatic filariasis in sub-Saharan Africa prior to scaling up interventions: integrated use of geostatistical and mathematical modelling. Parasites & Vectors. 2015;8(1):1–16. doi: 10.1186/s13071-015-1166-x 26496983 PMC4620019

[43] Ramaiah KD, Das PK, Michael E, Guyatt HL. The economic burden of lymphatic filariasis in India. Parasitology Today. 2000;16(6):251–253. doi: 10.1016/S0169-4758(00)01643-4 10827432

[44] Blower SM, Dowlatabadi H. Sensitivity and uncertainty analysis of complex models of disease transmission: an HIV model, as an example. International Statistical Review. 1994;62(2):229–243. doi: 10.2307/1403510

[45] Saltelli A, Tarantola S, Campolongo F, Ratto M. Sensitivity analysis in practice: a guide to assessing scientific models. vol. 1. Wiley Online Library; 2004.

[46] Marino S, Hogue IB, Ray CJ, Kirschner DE. A methodology for performing global uncertainty and sensitivity analysis in systems biology. Journal of Theoretical Biology. 2008;254(1):178–196. doi: 10.1016/j.jtbi.2008.04.011 18572196 PMC2570191

[47] Kirschner D. Uncertainty and sensitivity functions and implementation; 2020. http://malthus.micro.med.umich.edu/lab/usanalysis.html.

[48] Chu BK, Deming M, Biritwum NK, Bougma WR, Dorkenoo AM, El-Setouhy M, et al. Transmission assessment surveys (TAS) to define endpoints for lymphatic filariasis mass drug administration: a multicenter evaluation. PLoS Neglected Tropical Diseases. 2013;7(12):e2584. doi: 10.1371/journal.pntd.0002584 24340120 PMC3855047

[49] Toor J, Hamley JI, Fronterre C, Castaño MS, Chapman LA, Coffeng LE, et al. Strengthening data collection for neglected tropical diseases: What data are needed for models to better inform tailored intervention programmes? PLoS Neglected Tropical Diseases. 2021;15(5):e0009351. doi: 10.1371/journal.pntd.0009351 33983937 PMC8118349

[50] Geoffard PY, Philipson T. Disease eradication: private versus public vaccination. The American Economic Review. 1997;87(1):222–230.

[51] Acosta-Alonzo CB, Erovenko IV, Lancaster A, Oh H, Rychtář J, Taylor D. High endemic levels of typhoid fever in rural areas of Ghana may stem from optimal voluntary vaccination behaviour. Proceedings of the Royal Society A. 2020;476(2241):20200354. doi: 10.1098/rspa.2020.0354 33071586 PMC7544331

[52] Cheng E, Gambhirrao N, Patel R, Zhowandai A, Rychtář J, Taylor D. A game-theoretical analysis of Poliomyelitis vaccination. Journal of Theoretical Biology. 2020;499:110298. doi: 10.1016/j.jtbi.2020.110298 32371008

[53] Kobe J, Pritchard N, Short Z, Erovenko IV, Rychtář J, Rowell JT. A game-theoretic model of cholera with optimal personal protection strategies. Bulletin of Mathematical Biology. 2018;80(10):2580–2599. doi: 10.1007/s11538-018-0476-5 30203140

[54] Chouhan A, Maiwand S, Ngo M, Putalapattu V, Rychtář J, Taylor D. Game-theoretical model of retroactive Hepatitis B vaccination in China. Bulletin of Mathematical Biology. 2020;82(6):1–18. doi: 10.1007/s11538-020-00748-5 32542575

[55] Scheckelhoff K, Ejaz A, Erovenko IV, Rychtář J, Taylor D. Optimal Voluntary Vaccination of Adults and Adolescents Can Help Eradicate Hepatitis B in China. Games. 2021;12(4):82. doi: 10.3390/g12040082

[56] Broom M, Rychtář J, Spears-Gill T. The game-theoretical model of using insecticide-treated bed-nets to fight malaria. Applied Mathematics. 2016;7(09):852–860. doi: 10.4236/am.2016.79076

[57] Klein SRM, Foster AO, Feagins DA, Rowell JT, Erovenko IV. Optimal voluntary and mandatory insect repellent usage and emigration strategies to control the chikungunya outbreak on Reunion Island. PeerJ. 2020;8:e10151. doi: 10.7717/peerj.10151 33362952 PMC7750003

[58] Fortunato AK, Glasser CP, Watson JA, Lu Y, Rychtář J, Taylor D. Mathematical modelling of the use of insecticide-treated nets for elimination of visceral leishmaniasis in Bihar, India. Royal Society Open Science. 2021;8(6):201960. doi: 10.1098/rsos.201960 34234949 PMC8242840

[59] Brettin A, Rossi-Goldthorpe R, Weishaar K, Erovenko IV. Ebola could be eradicated through voluntary vaccination. Royal Society Open Science. 2018;5(1):171591. doi: 10.1098/rsos.171591 29410863 PMC5792940

[60] Ramaiah K, Kumar KV, Ramu K. Knowledge and beliefs about transmission, prevention and control of lymphatic filariasis in rural areas of South India. Tropical Medicine & International Health. 1996;1(4):433–438. doi: 10.1046/j.1365-3156.1996.d01-84.x 8765449

[61] Poletti P, Ajelli M, Merler S. The effect of risk perception on the 2009 H1N1 pandemic influenza dynamics. PloS One. 2011;6(2):e16460. doi: 10.1371/journal.pone.0016460 21326878 PMC3034726

[62] Xia S, Liu J. A computational approach to characterizing the impact of social influence on individuals’ vaccination decision making. PloS One. 2013;8(4):e60373. doi: 10.1371/journal.pone.0060373 23585835 PMC3621873

[63] Iwamura Y, Tanimoto J. Realistic decision-making processes in a vaccination game. Physica A: Statistical Mechanics and its Applications. 2018;494:236–241. doi: 10.1016/j.physa.2017.11.148

[64] Kabir KA, Jusup M, Tanimoto J. Behavioral incentives in a vaccination-dilemma setting with optional treatment. Physical Review E. 2019;100(6):062402. doi: 10.1103/PhysRevE.100.062402 31962423

[65] Kabir KA, Tanimoto J. Modelling and analysing the coexistence of dual dilemmas in the proactive vaccination game and retroactive treatment game in epidemic viral dynamics. Proceedings of the Royal Society A. 2019;475(2232):20190484. doi: 10.1098/rspa.2019.0484 31892836 PMC6936617

[66] Kuga K, Tanimoto J, Jusup M. To vaccinate or not to vaccinate: A comprehensive study of vaccination-subsidizing policies with multi-agent simulations and mean-field modeling. Journal of Theoretical Biology. 2019;469:107–126. doi: 10.1016/j.jtbi.2019.02.013 30807759

[67] Arefin MR, Masaki T, Kabir KA, Tanimoto J. Interplay between cost and effectiveness in influenza vaccine uptake: a vaccination game approach. Proceedings of the Royal Society A. 2019;475(2232):20190608. doi: 10.1098/rspa.2019.0608 31892839 PMC6936611

[68] Arefin MR, Kabir KA, Tanimoto J. A mean-field vaccination game scheme to analyze the effect of a single vaccination strategy on a two-strain epidemic spreading. Journal of Statistical Mechanics: Theory and Experiment. 2020;2020(3):033501. doi: 10.1088/1742-5468/ab74c6

[69] Kabir KA, Tanimoto J. Evolutionary game theory modelling to represent the behavioural dynamics of economic shutdowns and shield immunity in the COVID-19 pandemic. Royal Society Open Science. 2020;7(9):201095. doi: 10.1098/rsos.201095 33047059 PMC7540740

[70] Han CY, Issa H, Rychtář J, Taylor D, Umana N. A voluntary use of insecticide treated nets can stop the vector transmission of Chagas disease. PLoS Neglected Tropical Diseases. 2020;14(11):e0008833. doi: 10.1371/journal.pntd.0008833 33141850 PMC7671556

